# A Comprehensive COVID-19 Daily News and Medical Literature Briefing to Inform Health Care and Policy in New Mexico: Implementation Study

**DOI:** 10.2196/23845

**Published:** 2022-02-23

**Authors:** LynnMarie Jarratt, Jenny Situ, Rachel D King, Estefania Montanez Ramos, Hannah Groves, Ryen Ormesher, Melissa Cossé, Alyse Raboff, Avanika Mahajan, Jennifer Thompson, Randy F Ko, Samantha Paltrow-Krulwich, Allison Price, Ariel May-Ling Hurwitz, Timothy CampBell, Lauren T Epler, Fiona Nguyen, Emma Wolinsky, Morgan Edwards-Fligner, Jolene Lobo, Danielle Rivera, Jens Langsjoen, Lori Sloane, Ingrid Hendrix, Elly O Munde, Clinton O Onyango, Perez K Olewe, Samuel B Anyona, Alexandra V Yingling, Nicolas R Lauve, Praveen Kumar, Shawn Stoicu, Anastasiya Nestsiarovich, Cristian G Bologa, Tudor I Oprea, Kristine Tollestrup, Orrin B Myers, Mari Anixter, Douglas J Perkins, Christophe Gerard Lambert

**Affiliations:** 1 University of New Mexico School of Medicine Albuquerque, NM United States; 2 University of New Mexico Health Sciences Library and Informatics Center Albuquerque, NM United States; 3 University of New Mexico-Maseno Global Health Programs Laboratories Kisumu Kenya; 4 Department of Clinical Medicine, School of Health Sciences Kirinyaga University Kerugoya Kenya; 5 Department of Medical Biochemistry, School of Medicine Maseno University Maseno Kenya; 6 Center for Global Health, Division of Translational Informatics Department of Internal Medicine University of New Mexico Health Sciences Center Albuquerque, NM United States; 7 Department of Computer Science University of New Mexico Albuquerque, NM United States; 8 Health and Sciences Center Sponsored Projects Office University of New Mexico Albuquerque, NM United States; 9 Division of Translational Informatics Department of Internal Medicine University of New Mexico Health Sciences Center Albuquerque, NM United States; 10 University of New Mexico College of Population Health Albuquerque, NM United States; 11 New Mexico Department of Health Communications Office Office of the Secretary Santa Fe, NM United States

**Keywords:** COVID-19, pandemic, daily report, policy, epidemics, global health, SARS-CoV-2, New Mexico, medical education

## Abstract

**Background:**

On March 11, 2020, the New Mexico Governor declared a public health emergency in response to the COVID-19 pandemic. The New Mexico medical advisory team contacted University of New Mexico (UNM) faculty to form a team to consolidate growing information on severe acute respiratory syndrome coronavirus 2 (SARS-CoV-2) and its disease to facilitate New Mexico’s pandemic management. Thus, faculty, physicians, staff, graduate students, and medical students created the “UNM Global Health COVID-19 Intelligence Briefing.”

**Objective:**

In this paper, we sought to (1) share how to create an informative briefing to guide public policy and medical practice and manage information overload with rapidly evolving scientific evidence; (2) determine the qualitative usefulness of the briefing to its readers; and (3) determine the qualitative effect this project has had on virtual medical education.

**Methods:**

Microsoft Teams was used for manual and automated capture of COVID-19 articles and composition of briefings. Multilevel triaging saved impactful articles to be reviewed, and priority was placed on randomized controlled studies, meta-analyses, systematic reviews, practice guidelines, and information on health care and policy response to COVID-19. The finalized briefing was disseminated by email, a listserv, and posted on the UNM digital repository. A survey was sent to readers to determine briefing usefulness and whether it led to policy or medical practice changes. Medical students, unable to partake in direct patient care, proposed to the School of Medicine that involvement in the briefing should count as course credit, which was approved. The maintenance of medical student involvement in the briefings as well as this publication was led by medical students.

**Results:**

An average of 456 articles were assessed daily. The briefings reached approximately 1000 people by email and listserv directly, with an unknown amount of forwarding. Digital repository tracking showed 5047 downloads across 116 countries as of July 5, 2020. The survey found 108 (95%) of 114 participants gained relevant knowledge, 90 (79%) believed it decreased misinformation, 27 (24%) used the briefing as their primary source of information, and 90 (79%) forwarded it to colleagues. Specific and impactful public policy decisions were informed based on the briefing. Medical students reported that the project allowed them to improve on their scientific literature assessment, stay current on the pandemic, and serve their community.

**Conclusions:**

The COVID-19 briefings succeeded in informing and guiding New Mexico policy and clinical practice. The project received positive feedback from the community and was shown to decrease information burden and misinformation. The virtual platforms allowed for the continuation of medical education. Variability in subject matter expertise was addressed with training, standardized article selection criteria, and collaborative editing led by faculty.

## Introduction

On March 11, 2020, New Mexico Governor, Michelle Lujan Grisham, and the New Mexico Department of Health declared a public health emergency in response to the COVID-19 pandemic, after announcing 3 New Mexico residents tested presumptive positive for COVID-19 [1]. New Mexico Department of Health responded by creating the Medical Advisory Team, which brought together state officials, health care providers, and community members to compile and disseminate scientific findings, and create guidelines and recommendations to navigate the challenges of the pandemic. With the growing number of scientific publications and news reports and the potential for misinformation dissemination into the community, the Medical Advisory Team reached out to faculty at the University of New Mexico (UNM) to form a team to analyze and distribute reliable information to inform health care and public policy decisions for the state.

There was a high volume of both vital and inaccurate information available regarding COVID-19 [2,3]. To reduce information overload and misinformation, quality content had to be filtered and consolidated for state and health care leaders [4-6]. Researchers recommended the use of official public health organization websites as the most reliable source of information on COVID-19 preventative measures [7].

Prior to the COVID-19 pandemic, there was little guidance on methodology to generate comprehensive daily briefings. We aimed to provide guidance on how to create a daily briefing to address the high volume of information and misinformation. We surveyed the readers to determine if the briefing influenced their professional practice, if it was a main source of information, and if it helped combat misinformation. Readers also had the opportunity to share their thoughts on the briefings in free text.

During the first few months of the global pandemic, medical schools and various medical education governing bodies agreed that clinical medical education needed to be suspended due to high infectivity risk, limited COVID-19 testing supplies, and limited personal protective equipment (PPE). Studies have since assessed how psychologically and educationally detrimental it can be to study medicine in isolation. Participants in 1 study found an increase in depression, detachment from family and friends, and hopelessness, with a decrease in work performance and study time [8]. While virtual platforms are less ideal than in-person learning, learners found team-based projects to be more engaging [9,10]. Medical students were given the opportunity to take part in this project with the goal of increasing engagement during a difficult time of learning. We aimed to qualitatively determine if participation in the briefings showed net benefit to virtual medical education.

With a robust team of professionals, students, and volunteers, a comprehensive daily briefing was first disseminated on April 5, 2020, as the “UNM Global Health COVID-19 Intelligence Briefing.” Here, we describe the process of creating such briefings, the usefulness of these briefings to the community and its leaders, as well as their benefit to virtual medical education.

## Methods

### Briefing Process

**Figure 1:**
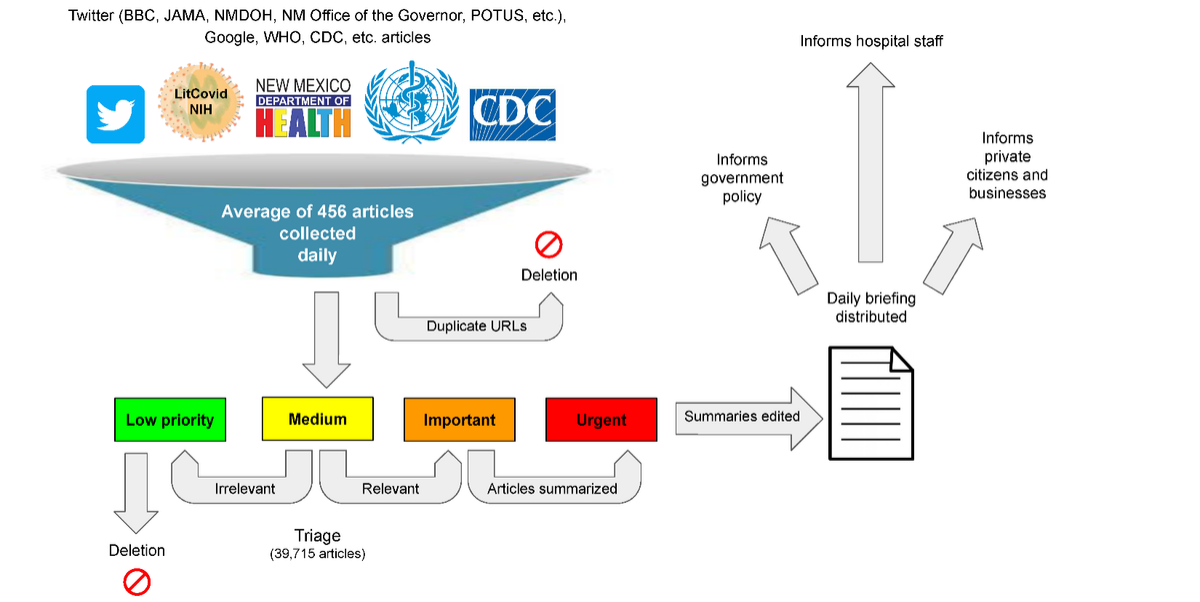
A conceptual overview of the methods used to generate the daily briefings. BBC: British Broadcasting Corporation; CDC: Centers for Disease Control and Prevention; JAMA: Journal of the American Medical Association; NMDOH: New Mexico Department of Health; POTUS: President of the United States; WHO: World Health Organization.

A team of medical doctors, PhDs, journalists, graduate students, medical students, and researchers from both the United States and Kenya volunteered to participate in the composition of the daily briefings. Microsoft Teams served as the platform for automatic and manual article collection and triaging, composition of the briefing, as well as the administration of a qualitative survey. Microsoft Teams Flows were developed to automatically gather COVID-19–related papers and reports from various sources including Twitter (eg, BBC, JAMA, New Mexico Department of Health, New Mexico Office of the Governor, President of the United States), Google, LitCovid (National Institutes of Health), World Health Organization, and the Centers for Disease Control and Prevention while excluding duplicate URLs (Figure 1; Multimedia Appendix 1). Articles were categorized into “New Mexico Mainstream Media,” “Manual Requests,” “US Mainstream Media,” “International Media,” “Health Organization,” “Science and Medicine,” and “Literature” and were assigned a default “medium” priority tag through Microsoft Planner. Articles manually submitted by readers through a submission link from each briefing generated a Microsoft Teams Planner task and were reviewed and triaged accordingly. Triaging was completed by 3 administrators to ensure quality and consistency.

Articles were manually triaged by administrators into “urgent,” “important,” or “low” priority groups. Scientific reports were triaged based on the veracity and potential impact on COVID-19–related health care or public policy response. Information on epidemiology, testing, public guidelines, medical practice guidelines, new therapies, vaccines, and pathogenesis were of primary focus. Evidentiary priority was given to systematic reviews of randomized controlled trials with meta-analyses and individual randomized controlled trials, followed by quasiexperimental, case control, or cohort studies. These were often labeled “urgent.” Studies with small sample sizes or problematic study designs were included or excluded at the triaging administrator’s judgment. Opinions pieces were largely excluded. News articles covering New Mexico, United States, and international pandemic responses and impacts were included based on relevance and content validity.

Articles labeled “important” were analyzed and summarized by a team member to include the type of study, pertinent results, other relevant information, the Digital Object Identifier, or hyperlink, and marked as “complete.” Headlines were carefully constructed to be as informative as possible rather than as “teasers.” Completed articles were autopopulated first into a Microsoft Excel spreadsheet, then relocated to a Microsoft Word document. Items typically triaged as “low” priority were commentaries, editorials, political messages, and non–evidence-based studies, and excluded from the briefing. The briefing was divided into the following sections: “Executive Summary;” “New Mexico Highlights;” “US Highlights;” “Economics, Workforce, Supply Chain, PPE Highlights;” “Epidemiology Highlights;” “Healthcare Policy Recommendations;” “Practice Guidelines;” “Testing;” “Drugs, Vaccines, Therapeutics, Clinical Trials;” and “Other Science.” Volunteers collaboratively edited the document with videoconference coordination each evening to ensure quality, clarity, accuracy, grammar, and proper citation. Reports with insufficient sample size, unclear conclusions, or poorly executed study designs were excluded. The final document included an “Executive Summary” with the general content, a disclaimer noting the inclusion of non–peer-reviewed content, and a list of all the participants of the day. The finalized briefing was emailed to the New Mexico Medical Advisory Team, sent to a listserv of subscribers, and posted on the publicly accessible UNM digital repository [11]. The UNM Department of Internal Medicine also incorporated the briefing into its daily department email. Listserv subscribers consisted of health care providers, researchers, and government employees, as well as UNM faculty, students, and readers not involved in health care or state policy. The UNM Digital Repository runs on BePress, which tracks global downloads of the briefings. The briefings have also been indexed by Google Scholar. There was no monetary gain associated with the creation of these briefings, with full access provided free of charge.

### COVID-19 Global Health Briefing Survey

To assess the usefulness of the briefings to New Mexico policy makers, researchers, educators, physicians, and other health care professionals, an 18-question survey was created through Microsoft Forms, approved by the Institutional Review Board (20-263), and distributed to all briefing recipients. Survey participant demographics included academic degree(s) and place of occupation. We determined if participants provided direct clinical care to COVID-19–positive patients, if the briefing was their primary source of information regarding the pandemic, and how likely they were to share the briefing with colleagues.

To determine the briefing’s usefulness to the respondent, we assessed the following on a scale from strongly disagree to strongly agree:

1. The daily briefing has informed or changed my response to the COVID-19 crisis.
2. I have gained relevant knowledge I wouldn’t have otherwise because of this briefing.
3. This briefing has enabled me to combat misinformation.
4. This briefing has enabled me to clarify information I have seen elsewhere.

Lastly, the respondent could respond in free text how information from the briefing was applied professionally or personally and if they had comments or suggestions to improve the briefings.

### Medical Education

Medical student education in the hospital setting was suspended, and students were not involved in direct patient care during the initial surge of COVID-19 around the globe. The UNM School of Medicine created a virtual COVID-19 course to allow for virtual didactics as well as asynchronous, self-directed learning. This course allowed students to be involved in various projects and, with curriculum committee approval, receive a 4th year elective credit for those efforts. A few students who were involved in the briefing proposed to the UNM School of Medicine curriculum committee that contribution to the briefings should count as course credit. The UNM School of Medicine approved the proposal.

Medical students were involved in manual submission of information, summarizing articles, editing the briefings every evening, and recruiting other students to join the project. The students also assisted in the assembly of the COVID-19 global health briefing survey. Lastly, the compilation and analysis of the data in this publication was entirely led by medical students who took part in various stages of the COVID-19 briefing development and dissemination.

## Results

### Briefing Process

Microsoft Teams Flows gathered an average of 456 articles daily, and 560 articles were manually submitted. A total of 39,715 articles were gathered throughout this project. Between April 5, 2020, to June 30, 2020, 58 UNM Global Health COVID-19 intelligence briefings were generated and published through the efforts of 68 individuals, including 19 faculty members, 31 medical students, 3 graduate students, 1 postdoctoral fellow, 3 staff members, and 11 contributors from outside of the UNM.

Each briefing was directly emailed to 176 members of the New Mexico Medical Advisory Team before the availability of the listserv on April 27, 2020. By June 30, 2020, the listserv had 400 subscribers. The UNM Department of Internal Medicine included the briefing in its daily newsletter sent to 525 members. By June 30, 2020, the briefing was sent to 1080 unique email addresses—693 within the UNM Health and Sciences Center and 387 outside of it.

**Figure 2:**
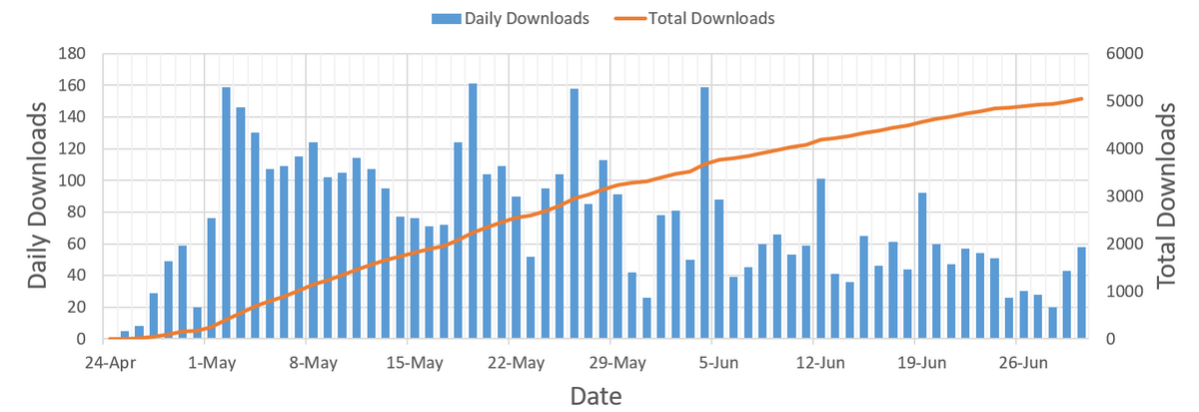
Bar graph showing downloads per day and total downloads over time.

Beginning April 24, 2020, all briefings were uploaded to the UNM Digital Repository for public access. The number of briefing downloads is plotted both daily and cumulatively in Figure 2. Between April 24, 2020, and July 5, 2020, there were 5047 downloads, with an average of 74 downloads per day. The highest number of downloads for an individual briefing was 260 (April 27, 2020). The average number of downloads was 85, the median 72, the maximum 260, and the minimum 4.

**Figure 3:**
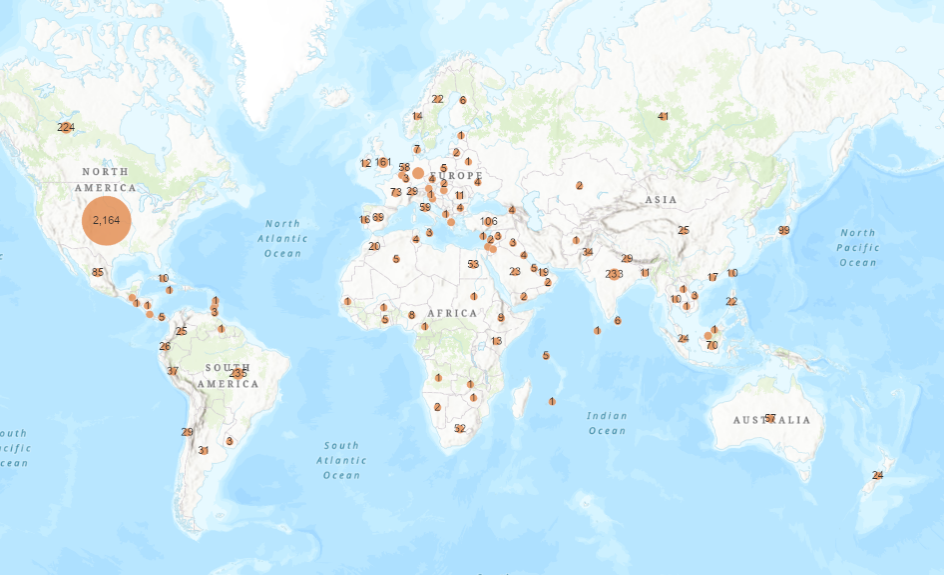
The number of downloads of University of New Mexico daily briefings per country from April 24, 2020, to July 5, 2020.

Throughout its entire duration, the briefings were downloaded in 116 countries (Figure 3). The 5 countries with the most downloads were the United States with 2164, Brazil with 235, India with 233, Canada with 224, and Germany with 224.

### COVID-19 Global Health Briefing Survey

To evaluate the usefulness of the briefings, we sent an electronic survey to approximately 994 readers. A total of 111 individuals (approximately 11%) responded between May 8 and May 19, 2020. Among the 111 responders, 41 (37%) were physicians, 41 (37%) nonclinical academic faculty, 9 (8%) administrators, 8 (7%) academic staff, 5 (5%) government employees, 4 (4%) students, and 3 (3%) nurses. Of the 111 respondents, 30 (27%) were involved in providing direct clinical care to COVID-19 patients, 47 (42%) had MD or DO degrees, 34 (31%) had PhDs, and 9 (8%) had MPH degrees. The health care providers who responded (n=30) had an average of 19 years of clinical practice (95% CI 15-23).

**Figure 4:**
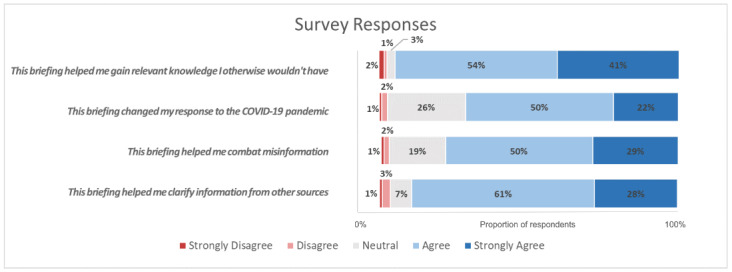
Survey responses from “strongly disagree” to “strongly agree”.

A majority of respondents (105/111, 95%) agreed or strongly agreed that the briefing helped them gain relevant knowledge, 71% (79/111) changed their response to the pandemic, 79% (88/111) reported the briefings helped them combat public misinformation, and 89% (99/111) said it helped to clarify the information from other data sources (Figure 4). Moreover, 24% (27/111) of respondents cited the briefing as their primary source of information on the pandemic, and 73% (81/111) reported having shared the briefing with their colleagues. On a scale of 0 (“would never share”) to 10 (“will definitely share”), the respondents reported being very likely to continue to share the briefing with colleagues (mean score 8.8, 95% CI 8.5-9.1).

The briefings influenced New Mexico state government response to the pandemic. David Scrase, the New Mexico Cabinet Secretary for Health and Human Services, informed our team that the daily briefing influenced dozens of policy decisions including the following: (1) mandating universal mask use in New Mexico early in the pandemic; (2) expansion of remdesivir treatment; (3) caution about hydroxychloroquine treatment; (4) selecting R_effective as a key gating criterion for the state (COVID spread rate); (5) guiding the adequacy of the PPE supply chain (particularly overseas); and (6) recommending against the use of antibody testing as an adjunct to clinical (or patient) decision making (David R Scrase email communication, June 16, 2020).

The briefings also received positive feedback from other health and policy officials around the state, including the UNM associate dean of Continuous Professional Learning, the Presbyterian chief medical and transformation officer, the vice chair of veterans’ affairs, the vice chancellor for clinical affairs, lead of New Mexico Medical Advisory Team, and the president and chief executive officer of Christus St. Vincent Regional Medical Center (Table 1).

**Table 1:** Direct quotes from health care and policy officials across New Mexico regarding the impact of the University of New Mexico briefings.

Author	Statement
Associate dean of Continuous Professional Learning at UNM^a^	“I recently became aware of the great work that you and colleagues are doing to cull through the infodemic to provide useful updates. I’d be grateful if you could add me to the distribution for the DAILY UNM GLOBAL HEALTH COVID-19 BRIEFING”
Presbyterian chief medical and transformation officer	“These are amazing! Can you help me get on the distribution list?”
Vice chair veteran’s affairs	“This compendium is excellent!”
Vice chancellor for clinical affairs, lead of New Mexico MAT^b^	“I wanted to let you know that you and the team know that your briefing is being sent to the PHS^c^ leadership team. It is getting rave reviews.”
Executive vice president and chancellor for HSC^d^; dean of School of Medicine	“You're doing a great job.” “Thank you and your team again...for this very comprehensive review of information. It is very useful.”
President and chief executive officer of Christus St. Vincent Regional Medical Center	“These are excellent briefs. Thank you for sharing.”
New Mexico cabinet secretary for Health and Human Services Department	“This is just so incredibly helpful… I really appreciate you taking the initiative to do this. Will provide daily highlights to gov and staff.”

^a^UNM: University of New Mexico.

^b^MAT: medical advisory team.

^c^PHS: public health service.

^d^HSC: Health and Sciences Center.

### Medical Education

The UNM Global Health COVID-19 Intelligence Report project enhanced medical student education. A total of 31 medical students contributed to the creation of the daily briefings. Participation in the project was approved for course credit by the UNM School of Medicine curriculum committee. The students were able to practice the analysis and quality assessment of scientific data, and they found participation in the briefings to be informative and rewarding (Textbox 1).

**Direct quotes from medical students regarding the impact of the briefings on medical education.**

**Medical students’ statements**

- “[The briefing] provided a meaningful purpose as we were able to contribute directly to our community’s wellness …. I appreciated participating, seeing daily updates, and knowing hospital teams were making real time policy changes with the help of the information provided in the reports.”
- “Once the COVID-19 block started it was refreshing to use my skills to analyze articles because I was already doing so on my own… It was great to be able to create deliverables as part of the project and feel like I was making an impact to the state of New Mexico, which eased my anxiety significantly.”
- “This style of project is immensely helpful for students who want to be more involved with research and have a limited history of working in research. I hope that every project I work on subsequently will be so well organized.”
- “Participating in the daily briefing helped me stay connected to the ever-changing world of COVID research. In a world of information overload, helping with this project grounded me to the primary information coming out of the scientific world. I felt confident giving friends and family advice and educating them on this pandemic because of the briefing.”
- “Being involved in this project has helped me learn more about this novel virus and feel more confident about the information I can disseminate to my friends and family. It also taught me about how difficult it can be to find trustworthy news, and how much of a problem can misinformation be. Even my parents (who are college educated with scientific degrees) presented wrong information to me many times. This reminded me of the importance of reliable news sources these days, and how we struggle to find them.”

## Discussion

### Strengths and Principal Findings

The development of the briefing succeeded in informing the practice of physicians and other health care personnel, facilitating COVID-19 research efforts by faculty and staff in academia, influencing policy making by the government of New Mexico, and alerting the public about the most relevant developments as is evident in our survey results. Additionally, the briefings were accessed by approximately 3 times the number of people compared with those who were directly emailed and catered to an audience beyond the scientific and medical community as the categorization of articles with headers and summaries reported in everyday language.

This project also succeeded in allowing for the continuation of medical education during the initial surge of COVID-19 when medical students and research students were prohibited from their normal duties. The learners improved on their ability to analyze a scientific paper and helped them empathize with the public regarding misinformation. The students reported being part of a community project to be meaningful and that influencing the health of New Mexico reduced student anxiety of being away from direct patient care.

The utilization of a listserv and online archive helped expand the audience beyond medical advisory team and the state of New Mexico to the briefing's eventual global reach. Organizing interest through a listserv would help disseminate the material quicker and broader should another similar endeavor be attempted.

Due to both limited funding and volunteers, the effort was brought to a close as the final briefing was published on June 30, 2020. We were unable to continue the production of thorough, high-quality publications free of charge due to the inevitable loss of many of our volunteers. We were unable to replace team administrators and leadership to triage daily and ensure quality control. Medical students returned to clinical duties, and faculty were unable to simultaneously sustain this unfunded effort along with their teaching, research, and administrative responsibilities. Between April 9, 2020, to July 2, 2021, a similar COVID-19 briefing effort was created (“COVID-19 Literature Surveillance Team”) of medical students and faculty, who were able to continue their reports by changing their daily briefing to weekly [12]; however, this team ultimately discontinued publication [13]. Our COVID-19 briefing efforts were closed with a presentation that expressed gratitude to all who participated [14].

### Limitations

Contributors and authors of the briefings had variable expertise in assessing scientific literature. Initially, all participants were allowed to triage content. Due to quality concerns, triaging was reduced to 3 administrators. It was not obvious how to standardize the judgment process that went into rapid decision-making regarding article inclusion. Those triaging needed to be aware of the prior body of work addressed, be able to analyze an article quickly, and be well versed in various research methodologies and their assessment. Standardized training for editors and authors was implemented to improve the consistency and quality of article summaries.

Initial article selection was less stringent and included more speculative reports. More selective criteria were implemented over time. The inclusion of articles of varying evidence, such as preprints, received criticism. Recent studies have shown that the standard in preprints for the life sciences is similar to that of peer-reviewed articles; therefore, they can be considered valid scientific contributions [15]. As a clarification, our later briefings included the source and type of study (preprint, meta-analysis, Reuters, etc) before each summary.

A major concern was the disagreement between global governing entities on treatment and the quickly evolving understanding of the virus. For example, the US Food and Drug Administration granted emergency use authorization for both chloroquine and hydroxychloroquine for certain hospitalized COVID-19 patients on March 28, 2020, whereas the European Union did not allow for its use. The emergency use authorization was revoked on June 15, 2020, due to side effects of the medications.

While our survey was disseminated to all subscribers of the briefing, persons who may have derived less value from the briefings or had more substantial criticism of the publication were not well represented among the respondents. While favorable impact ratings were provided by survey respondents, our free-text section (on how the respondent-applied information from the briefing influenced their active response to the pandemic) could have benefitted from more specific wording to solicit specific uses. The most substantive feedback came from a nonsurvey source, namely Secretary Scrase via email.

### Comparison to Prior Work and Future Directions

Other programs have released daily briefings [16,17,18,19,20] in similar efforts to combat information overload and implement quality control during the COVID-19 pandemic. At the time of this publication, there has been no research that has compared briefing dissemination to other forms of information dissemination during a rapidly changing health crisis. The pandemic has shifted the scientific community toward a more robust and rapid data-sharing culture [21]. Future work could compare the efficacy of briefings compared to other measures to alter and improve policy and information dissemination.

## Appendix

Multimedia Appendix 1 Data accumulation.

## Abbreviations

PPE: personal protective equipment
UNM: University of New Mexico
