# Supplementary material for: A Comprehensive COVID-19 Daily News and Medical Literature Briefing to Inform Health Care and Policy in New Mexico: Implementation Study
Source: JMIR Med Educ. 2022 Feb 23;8(1):e23845. doi: 10.2196/23845 (PMC8908195; doi:10.2196/23845)
Supplement: Multimedia Appendix 1 [file mededu_v8i1e23845_app1.docx]

**Multimedia Appendix 1: Data Accumulation**

Numerous Microsoft Teams™ Flows were set up to gather data from various sources with keywords. These reports were auto-populated into Microsoft Teams™ Planner as tasks to be further triaged. The following represent the automated literature searches.

Google Alert RSS Feed

"new mexico" AND (coronavirus | covid-19 | SARS-CoV-2 | governor)

(coronavirus | covid-19 | SARS-CoV-2) site:fda.gov

(hydroxychloroquine | "coronavirus" | "covid-19" | "SARS-CoV-2") site:medrxiv.org

(hydroxychloroquine | "coronavirus" | "covid-19" | "SARS-CoV-2") site:unm.edu

Other RSS Feeds

LitCovid: <https://www.ncbi.nlm.nih.gov/research/coronavirus-api/feed/?filters=%7B%7D>

Reuters Health: <http://feeds.reuters.com/reuters/healthNews>

Twitter Sources

(#covid19 OR #coronavirus OR #sarscov2) ((from:JAMANetwork) OR (from:JAMA_current)) -filter:retweets

(from:WHO) filter:links -filter:replies -filter:retweets since: formatDateTime(addDays(utcNow(),-2),'yyyy-MM-dd')

((coronavirus OR "covid-19" OR (#coronavirus) OR (#covid19)) (from:BBCWorld) -filter:retweets

from:@CDCgov -filter:retweets

((coronavirus OR "covid-19" OR (#coronavirus) OR (#covid19)) (from:AJEnglish) -filter:retweets

from:@NMDOH -filter:retweets

(#covid19 OR #coronavirus OR #sarscov2) ((from:ScienceMagazine) OR (from:NewsfromScience) OR (from:nresearchnews) OR (from:nature) OR (from:NEJM)) -filter:retweets

from:@realDonaldTrump -filter:retweets

from:@GovMLG
